# Enhanced Neutralization Potency of Botulinum Neurotoxin Antibodies Using a Red Blood Cell-Targeting Fusion Protein

**DOI:** 10.1371/journal.pone.0017491

**Published:** 2011-03-02

**Authors:** Sharad P. Adekar, Andrew T. Segan, Cindy Chen, Rodney Bermudez, M. D. Elias, Bernard H. Selling, B. P. Kapadnis, Lance L. Simpson, Paul M. Simon, Scott K. Dessain

**Affiliations:** 1 Lankenau Institute for Medical Research, Wynnewood, Pennsylvania, United States of America; 2 Immunome, Inc., Wynnewood, Pennsylvania, United States of America; 3 Division of Infectious Diseases and Environmental Medicine, Thomas Jefferson University, Philadelphia, Pennsylvania, United States of America; 4 Impact Biologicals, Inc. Wallingford, Pennsylvania, United States of America; 5 Department of Microbiology, University of Pune, Pune, India; 6 Augmenta Biologicals, LLC, Wynnewood, Pennsylvania, United States of America; Institut de Pharmacologie et de Biologie Structurale, France

## Abstract

Botulinum neurotoxin (BoNT) potently inhibits cholinergic signaling at the neuromuscular junction. The ideal countermeasures for BoNT exposure are monoclonal antibodies or BoNT antisera, which form BoNT-containing immune complexes that are rapidly cleared from the general circulation. Clearance of opsonized toxins may involve complement receptor-mediated immunoadherence to red blood cells (RBC) in primates or to platelets in rodents. Methods of enhancing immunoadherence of BoNT-specific antibodies may increase their potency *in vivo*. We designed a novel fusion protein (FP) to link biotinylated molecules to glycophorin A (GPA) on the RBC surface. The FP consists of an scFv specific for murine GPA fused to streptavidin. FP:mAb:BoNT complexes bound specifically to the RBC surface *in vitro*. In a mouse model of BoNT neutralization, the FP increased the potency of single and double antibody combinations in BoNT neutralization. A combination of two antibodies with the FP gave complete neutralization of 5,000 LD50 BoNT in mice. Neutralization *in vivo* was dependent on biotinylation of both antibodies and correlated with a reduction of plasma BoNT levels. In a post-exposure model of intoxication, FP:mAb complexes gave complete protection from a lethal BoNT/A1 dose when administered within 2 hours of toxin exposure. In a pre-exposure prophylaxis model, mice were fully protected for 72 hours following administration of the FP:mAb complex. These results demonstrate that RBC-targeted immunoadherence through the FP is a potent enhancer of BoNT neutralization by antibodies *in vivo*.

## Introduction

Botulinum neurotoxin is one of the most potent lethal substances known. It is produced by organisms of the genus *Clostridium* and produces peripheral neuromuscular and autonomic paralysis through inactivation of cholinergic signaling at the neuromuscular synapse. Intoxication with BoNT proceeds by a series of steps, in which BoNT first enters the body, transits across an epithelium, travels through the bloodstream, and interacts with the surface of cholinergic neurons [1], [2], [3]. Once bound to the neuromuscular junction, BoNT is internalized via binding to secretory vesicle proteins and transported into a vesicular compartment. The catalytic domain of BoNT, the light chain (LC), acquires proteolytic activity as it is transported across the vesicle membrane into the neuron cytosol [4], [5]. Through cleavage of tethering proteins, the BoNT LC prevents the neuron from releasing acetylcholine in response to neural stimulation.

Passive immune therapies for BoNT intoxication have been shown to be effective clinically and in laboratory studies, with either antisera or oligoclonal combinations of monoclonal antibodies [6], [7], [8]. Within the bloodstream, BoNT-containing immune complexes that contain three or more antibodies are rapidly sequestered in the spleen and liver [3], [8]. Such clearance is sufficient to provide high level neutralization (>10,000 LD50 BoNT), even if the antibodies do not have intrinsic neutralizing activity [9], [10]. Immune complexes formed between BoNT and only one or two antibodies stably circulate in the bloodstream and are therefore much less potent in BoNT neutralization (L.L.S., data not shown).

A general feature of the handling of immune complexes *in vivo* is immunoadherence, i.e., attachment to red blood cells (RBC) [11]. The precise mechanism for BoNT clearance by immune complexes has not been elucidated, but it may involve multiple, redundant systems for antigen capture by Fcγ receptor-bearing reticuloendothelial cells in the liver and spleen [8], [12], [13]. One aspect of this process utilizes the complement system, in which C3b-opsonized immune complexes bind to complement receptor type 1 (CR1) on RBCs in primates or to complement factor H in rodents [14], [15]. The ability of a monoclonal antibody to utilize this pathway can be enhanced by linking it to another antibody specific for CR1, to create a bispecific “heteropolymer” [16], [17]. Heteropolymer:antigen complexes bound to RBCs can be directly taken up by macrophages and are rapidly cleared from the circulation.

Methods that enhance the immunoadherence of antibodies to RBCs may be useful for BoNT prophylaxis and treatment. Antibody immunoadherence may be enhanced using a novel fusion protein (FP), created by Augmenta Biologicals (Wynnewood, PA). The FP is a recombinant protein that links streptavidin [18] to an scFv derived from a monoclonal antibody specific for GPA, the predominant protein on the RBC surface [19]. The FP was developed as a delivery system to adhere biotinylated molecules to the RBC surface, which may enhance the immunogenicity of biotinylated vaccine antigens and the clearance of biotinylated antibody-antigen complexes. We previously described a panel of human monoclonal antibodies specific for BoNT serotypes A and B (BoNT/A, BoNT/B) [20], [21], [22]. In this study, we examined the ability of the FP to augment the neutralizing capability of these antibodies *in vivo*.

## Results

### Binding of FP:mAb:BoNT complexes to red blood cells *in vitro*

**Figure 1:**
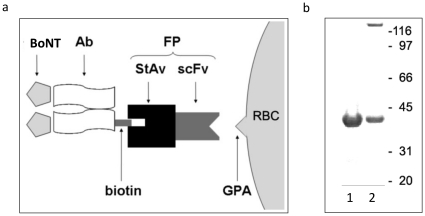
The RBC-targeting fusion protein (FP). (a) Schematic representation of the FP. The FP is comprised of an scFv, specific for the RBC surface protein glycophorin A (GPA), fused to streptavidin (StAv). The latter is capable of binding biotinylated mAbs specific for BoNT. (b) SDS-PAGE of the FP performed without heating the samples prior to loading. Lane 1: FP after expression in *E. coli* and purification in 8M urea (monomer). Lane 2: refolded FP following dialysis for removal of urea showing the tetramer and residual monomer.

The FP is a recombinant protein that joins an N-terminal scFv specific for GPA to a C-terminal streptavidin moiety (**Figure 1a**). GPA is expressed exclusively on the RBC membrane, at approximately 10^6^ copies per cell, where its primary role is to provide negatively charged sialic acid residues that limit RBC-RBC aggregation [19]. The murine scFv sequence specific for GPA was derived from the antibody TER-119 [23]. Streptavidin is a tetrameric protein that binds biotin with high affinity [24]. The FP also contains a C-terminal polyhistidine tag to facilitate purification following expression in *E. coli*. **Figure 1b** shows a poly-acrylamide gel in which urea-solubilized and refolded FP samples were analyzed. Two bands were seen in the refolded FP sample, consistent with existence of tetrameric (164 kDa) and monomeric (41 kDa) forms. We have observed that the monomeric FP is unable to bind biotin (P.M.S., data not shown), consistent with the observations of others [24].

**Figure 2:**
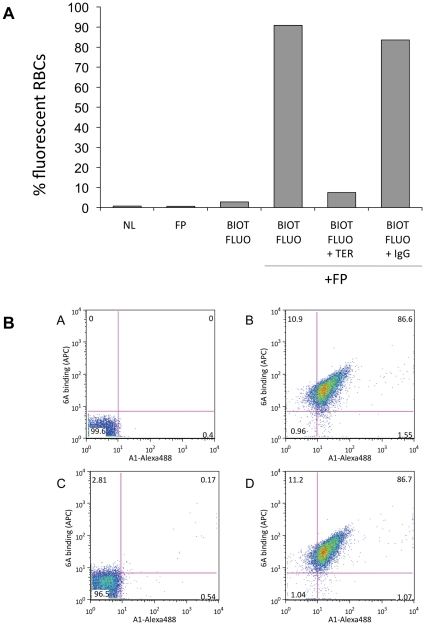
Binding of FP and FP:mAb complexes to GPA on murine RBCs. (a) *In-vitro* RBC binding by the FP complex is specific for glycophorin A. FP with or without biotinylated-fluorescein (BIOT-FLUO) was incubated with excess competitor TER-119 antibody (TER) or an isotype control (IgG) and analyzed by FACS. NL, no label. (b) RBC binding of the FP:6A:BoNT complex. A) Unlabeled RBCs. B) FP, biotinylated 6A and Alexa488-labeled HC50A were added to RBCs and detected with an anti-human-APC antibody. C) Competitor TER-119 inhibited binding of the complex to RBCs. D) An IgG isotype control antibody did not affect complex binding.

As depicted in **Figure 1a**, the FP was designed as a molecular bridge to link biotinylated molecules, such as antigens and antibodies, to the RBC membrane. We analyzed binding of the FP to the surface membrane of murine RBCs *in vitro* using flow cytometry, labeling the FP with biotinylated fluorescein. **Figure 2a** shows near complete labeling of the RBCs mediated by the FP molecule. FP binding was specific for GPA, since its binding was completely inhibited by the TER-119 IgG, but not by an isotype control antibody (rat IgG2b). Next, we tested RBC binding of complexes containing FP, the BoNT/A-specific MAb 6A, and BoNT/A 50 kDa C-terminal domain (HC50). The HC50 was labeled with Alexa Fluor 488, and the biotinylated 6A MAb was detected with an anti-human IgG-APC secondary antibody. *In vitro* incubation of this complex with RBCs resulted in almost complete co-labeling of RBCs with APC and Alexa-488 (**Figure 2b** **, panels A and B**). This binding was also inhibited by TER-119, but not by the isotype control antibody (**Figure 2b** **, panels C and D**).

### *In vivo* neutralizing ability of single antibodies bound to toxin

We previously reported three human antibodies that are specific for BoNT [20], [21]. 4LCA binds to the catalytic light chain domain of BoNT/A, and can neutralize 25 LD50 BoNT/A in the standard mouse protection assay. The 6A and 13A mAbs bind to overlapping epitopes on the BoNT/A heavy chain C-terminal 50 kDa domain (HC50). The 6A MAb can neutralize 2.5 LD50 BoNT/A *in vivo*, while the 13A MAb is essentially non-neutralizing. The 30B MAb binds serotype B BoNT (BoNT/B) with high affinity, but it is not neutralizing. We biotinylated these mAbs and tested them *in vivo* alone or in combination with excess FP. The most effective complex contained 6 µg FP and 1.5 µg 4LCA, which was able to completely neutralize up to 100 LD50 BoNT, 4-fold greater than could be neutralized by 100 µg naked 4LCA MAb (**Table 1**). The FP also enhanced the activity of the 6A MAb, allowing neutralization of 25 LD50 BoNT/A, a 10-fold increase over the 2.5 LD50 neutralized by 100 µg naked 6A. Control experiments with un-biotinylated 4LCA and 6A mAbs, administered with FP, showed no enhancement in activity (data not shown). Incorporated into an FP complex, the non-neutralizing 13A MAb protected mice up to a dose of 10 LD50. Similarly, the non-neutralizing 30B MAb could neutralize BoNT/B *in vivo* when bound to the FP, although the dose of complex was larger (12 µg FP, 3 µg 30B) and the amount of toxin was smaller (5 LD50). These observations indicate that the FP can enhance the neutralizing activity of BoNT mAbs. In addition, while FP quantitatively enhanced the potency of mAbs with intrinsic neutralizing activity, it also converted qualitatively non-neutralizing mAbs to neutralizing ones.

**Table 1:** Neutralization of BoNT by single mAbs in combination with FP.

Antibody	mAb µg	BoNT serotype	FP µg	2.5 LD50 % alive	5 LD50 % alive	10 LD50 % alive	25 LD50 % alive	100 LD50 % alive	250 LD50 % alive
13A	100	A	0	0		0			
13A	1.5	A	6	100		100	0		
6A	100	A	0	100		0	0	0	
6A	1.5	A	6	100		100	100	0	
4LCA	100	A	0	100		100	100	0	0
4LCA	1.5	A	6	100		100	100	100	66
30B	100	B	0		0	0			
30B	3	B	12		100	20			

Antibodies and FP:mAb complexes were tested for their ability to protect mice from lethality induced by botulinum neurotoxin (BoNT). mAbs were tested alone, without modification, or biotinylated and in combination with the fusion protein (FP) by mixing with the toxin *in vitro* and intravenous injection one hour later. Mice were observed for 5 days. The amounts of mAb and FP used per mouse (µg), the serotype of each BoNT (A or B), and the percent of surviving mice for each dose (LD50) administered are shown. Blank spaces indicate dose levels that were not tested.

### FP:mAb neutralization with MAb combinations and reduction in plasma BoNT concentration

A recurrent finding of BoNT neutralization *in vivo* is that oligoclonal antibody mixtures are more potent than single antibodies. We tested a combination of the 4LCA and 6A mAbs, which together, in their unmodified forms, can neutralize up to 1000 LD50 [22]. Using 0.75 µg each MAb with 6 µg FP, we observed complete survival with up to 2000 LD50 BoNT, whereas 50 µg each of naked 4LCA and 6A was not protective (**Table 2**). Increasing the quantity of FP:Ab 4-fold (24 µg FP with 3 µg each MAb), provided complete survival with BoNT doses of up to 5000 LD50. Mice that received this dose and 10,000 LD50 BoNT/A survived one day following the injection, indicating partial protection.

**Table 2:** Neutralization of BoNT *in vivo* with combinations of the 4LCA and 6A mAbs and the FP.

4LCA Biotin µg	4LCA µg	6A Biotin µg	6A µg	FP µg	SA µg	2,000 LD50 % Survival
	50		50	0		0
0.75		0.75		6		100
	1.5	1.5		6		0
	100	1.5		6		0
0.75		0.75			2	0
0.75		0.75			6	0

The 6A and 4LCA mAbs were tested in un-modified and biotinylated forms, alone and in combination with the fusion protein (FP) or streptavidin (SA). The combinations were tested by mixing and incubation *in vitro*, with 2,000 LD50 BoNT/A, followed by intravenous injection. The doses of each component are given in µg, and the outcomes are reported as the percentage of mice surviving (% Survival).

**Figure 3:**
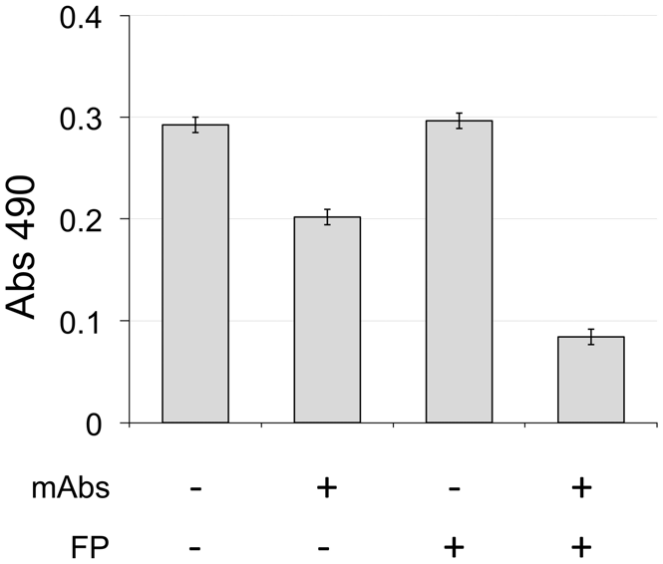
Plasma BoNT/A concentrations in mice injected with biotinylated anti-BoNT antibodies with or without FP. Groups of three mice each were injected i.v. with 6 µg of inactivated BoNT/A, in the presence of the indicated combinations of biotinylated mAbs 6A and 4LCA (3 µg each) and FP (24 µg). After 90 minutes, blood was collected and plasma BoNT/A concentrations were determined by ELISA.

We next used the 2000 LD50 dose to test the importance of linking the MAb to the RBC surface through the FP. Unbiotinylated 4LCA, at either 1.5 µg or 100 µg, did not contribute to neutralization by biotinylated 6A and the FP (**Table 2**). When both mAbs were biotinylated, but given in combination with streptavidin, rather than FP, no protection was seen. These results suggest that efficacy *in vivo* requires the formation of a complex that anchors both mAbs to the RBC surface by binding to one or more FP molecules. To explore this further, we assessed the plasma BoNT levels in mice injected with 6 µg of detoxified BoNT/A and the 4LCA and 6A FP:mAb complexes (3 µg each antibody, 24 µg FP) (**Figure 3**). Ninety minutes after injection, we tested the levels of BoNT/A in undiluted plasma using ELISA. The presence of the biotinylated mAbs alone reduced the BoNT levels slightly, but this effect was significantly enhanced by the presence of the FP. Taken together, these results demonstrate the collaborative effects of a pair of mAbs in combination with the FP, and suggests that the neutralizing activity requires BoNT immunoadherence to RBCs *in vivo*.

### Post-exposure and pre-exposure neutralization of BoNT by FP:mAb complexes

**Figure 4:**
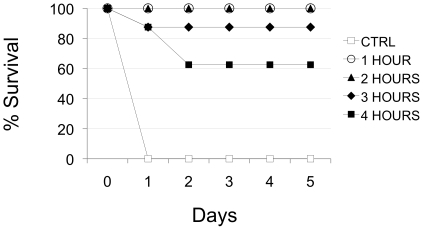
Post-exposure injection of an FP:mAbs combination protects mice from BoNT/A lethality. BoNT/A (10 LD50) was administered via intra-peritoneal injection to Swiss Webster mice, which subsequently received an intravenous injection of biotinylated mAbs 6A and 4LCA (0.75 µg of each) and 6 µg FP at either 1 hour (clear circles), 2 hours (triangles), 3 hours (diamonds), or 4 hours (squares) following the BoNT/A dose. Control mice (clear squares) received BoNT/A only. The FP:mAbs complex provided 100% protection at 1 hour and 2 hours post-BoNT, and 87.5% and 62.5% protection at 3 and 4 hours post-BoNT, respectively.

**Figure 5:**
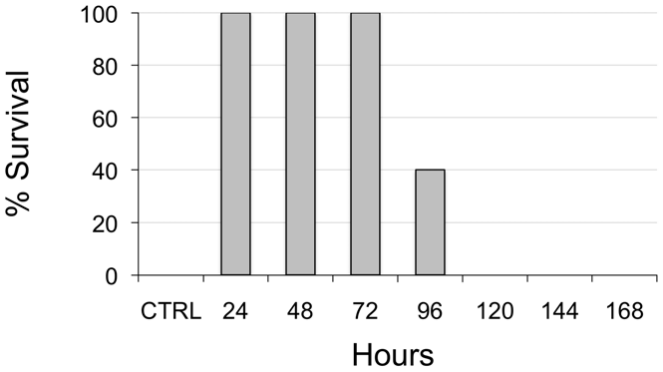
Pre-exposure prophylaxis of BoNT/A lethality with an FP:mAbs combination. Mice received i.v biotinylated mAbs 6A and 4LCA (0.75 µg of each) and 6 µg FP. They were subsequently challenged with 10 LD50 BoNT i.p. at the indicated times. Data are plotted as the percent of mice surviving 5 days after the BoNT exposure. All mice that received the FP:mAbs combination up to 72 hours prior to BoNT survived. Mice given BoNT 96 hours after the FP:mAbs combination showed partial protection (40% survival).

We next assessed the FP:Ab mixture in post-exposure and pre-exposure models, testing 6 µg FP with 0.75 µg each of the 4LCA and 6A antibodies. In the post-exposure model, BoNT (10 LD50) was delivered by intraperitoneal (i.p.) injection and FP:mAb complexes were subsequently administered at hourly intervals by intravenous (i.v.) injection. Mice were monitored for survival for 5 days. Complete survival was provided by FP:Ab given up to 2 hours following the BoNT injection, with partial survival at 3 and 4 hours post-BoNT (87.5% and 62.5% survival, respectively; **Figure 4**). We also performed a pre-exposure challenge with this dose of FP:mAb. Mice first received an i.v. injection of the FP:mAb and were then challenged at daily intervals with 10 LD50 BoNT/A i.p. (**Figure 5**). FP:mAb complexes were able to provide complete survival for mice receiving BoNT up to 72 hours after FP:mAb administration. Mice receiving BoNT 96 hours after the FP:mAb were partially protected (40% survival). Thus, the FP:mAb combination can provide protection against a lethal dose of BoNT in both post-exposure and pre-exposure conditions.

## Discussion

The primary obstacles to the development of a comprehensive immune therapeutic for BoNT exposure are the extreme potency of the toxin and the broad diversity of BoNT serotypes. Experiments with polyclonal antibodies and oligoclonal antibody combinations have shown that effective clearance of BoNT from the circulation is one of the most important contributors to neutralization [1], [3], [8], [22], [25], [26]. The development of single or double molecule therapeutics for BoNT will require enhancement of the ability of mAbs to clear BoNT from the blood circulation.

In this study, we tested the ability of a novel FP to enhance the neutralizing capacity of BoNT-specific mAbs at the level of the blood circulation. The FP is a bifunctional molecule that allows the adherence of biotin-conjugated immune complexes to the RBC surface. The FP was able to significantly increase the neutralization potency of BoNT-specific mAbs. When combined with the FP, the non-neutralizing 13A and 30B mAbs were able to fully neutralize lethal doses of BoNT/A and BoNT/B, respectively. The FP increased the quantity of BoNT/A that could be neutralized by the 6A mAb, the 4LCA mAb, and the 6A/4LCA mAb combination. These levels were achieved with much lower quantities of mAbs than we had previously required for protection with naked mAbs (1.5 µg vs. 50-100 µg). The NIH and the FDA have not yet established a standard for protective efficacy for BoNT passive immune therapies, but we can compare the potency of our FP combinations with the approved BoNT anti-toxins. The human antiserum for use in infants, BabyBig (MassBioLogics, Boston, MA), has a BoNT/A-specific potency of 3,000 LD50/mg [27]. Two of our single FP:mAb combinations (with 6A or 4LCA) and our double MAb combination (with both 6A and 4LCA) were equivalent or better in potency (3,333–266,666 LD50/mg, **Table 3**). The investigational heptavalent botulinum antitoxin (HBAT, Cangene Corporation, Winnipeg, Manitoba, CA) has a potency of 7,500 IU/dose (1 IU equals 10,000 murine LD50s) [28]. An equivalent dose of the FP:mAb combination would amount to 281 mg, or 4 mg/kg in a 70 kg person. Thus, the potency of our FP:mAb combination is within the range required for a human therapeutic.

**Table 3:** Comparative potencies of antibody-based BoNT therapeutics.

Antibody	LD50/mg* no FP	LD50/mg** with FP	Ratio***
13A	0	1,333	N/A
6A	25	3,333	133
4LCA	250	13,333	53
6A+4LCA	10,000	266,666	26.6
BabyBig [27]	3,000		

Comparative potencies were calculated using data from this study and the published Summary Statement of Approval for BabyBig [27].

*100 µg mAb administered.

**1.5 µg mAb with 6 µg FP administered.

***Calculated as: LD50/mg (FP:mAb)/LD50/mg (mAb alone).

Animal testing of a BoNT countermeasure needs to address protection of an asymptomatic individual (who has absorbed a sub-lethal dose, but may still be absorbing toxin) or an individual at risk for exposure (such as a first responder to a contaminated area). We found that the FP combined with 6A and 4LCA is sufficient to provide neutralization of 10 LD50 BoNT/A for 72 hours and partial protection at 96 hours. This result indicates the presence of physiologically relevant concentrations of the FP:mAb combination *in vivo* during this time.

We also tested the FP:mAb combination in a post-exposure model, in which the FP:Ab was administered following a lethal dose of toxin. An intravenous injection of the combination was able to provide complete protection from a lethal intraperitoneal BoNT dose at 2 hours and partial protection at 4 hours. BoNT distribution experiments have demonstrated that the window of opportunity of exposure of a lethal dose of BoNT is determined by a first-order reaction that depends on the amount of BoNT administered and the period of time that elapses before the countermeasure is administered (L.L.S., manuscript in preparation). Thus, anti-toxins that are sufficient to neutralize an entire circulating dose of BoNT should give the same window of opportunity for post-exposure salvage, and our post-exposure results are comparable to results obtained by others [10], [28], [29].

The proposed mechanism through which the FP augments antibody neutralization activity involves binding of FP:mAb complexes to the RBC surface. Flow cytometry experiments *in vitro* demonstrated that the FP:mAb complexes are able to bind to erythrocytes and that the complexes serve as a molecular bridge to adhere BoNT to the RBC. Experiments with un-biotinylated antibodies and the FP did not show any enhancement of neutralization. Streptavidin alone did not improve neutralizing activity of biotinylated BoNT antibodies, in comparison to the RBC-targeted FP. Lastly, the levels of BoNT circulating in the plasma of mice that had received the FP in combination with the 4LCA and 6A were lowered. These results together support the model that the enhancement of neutralization *in vivo* required biotin-dependent interaction of the cloned mAbs with the FP and adherence of the FP-containing complexes to the surface of the RBCs (**Figure 1a**).

The observation that the intrinsic neutralization capacity of the naked mAbs correlated directly with the potency level achieved when bound to the FP supports the idea that the FP:mAb:BoNT complexes remain in circulation for a significant period of time before they are definitively removed. In this model, relatively rapid adherence of FP:mAb:BoNT to the RBC membrane would be followed by a slower phase, in which either complex-bound toxin is removed from the RBC surface or the BoNT-bound RBCs are removed from the circulation.

This is distinct from clearance of C3b-opsonized immune complexes, which are definitively taken up by the liver and spleen in less than 15 minutes [8], [13]. While circulating and adherent to RBCs, the FP:mAb complexes would be in competition with the neuromuscular junction for BoNT. Intoxication may result from dissociation of BoNT from the antibody complex, or of the FP:mAb:BoNT complex from the surface of the RBC. The high potency of the FP:6A/4LCA complex may partly result from stabilization of BoNT on the RBC surface through cooperative mAb avidity effects, as maximal neutralization with the 4LCA and 6A antibodies was only observed when both were biotinylated. Accelerated RBC destruction is not likely to be a factor in BoNT clearance, as mice treated with FP do not exhibit a reduced hematocrit (data not shown).

Our study has shown the value of immunoadherence as an effective mechanism for improving the neutralizing ability of BoNT mAbs. The potency of the FP:mAb complexes and their utility in the pre-exposure setting demonstrated that immunoadherent immune complexes could be used to protect those at risk of BoNT exposure, in addition to those already exposed. In practice, the FP could be held in a biodefense stockpile as a non-specific immune adjuvant, to be combined with biotinylated MAb specific for the toxin(s) to which people have been exposed. Alternatively, FP sequences could be used to create hybrid MAb molecules that combine, in a single polypeptide, RBC immunoadherent and anti-toxin activities in a single construct. An important advantage of the FP is that it can be ligated quickly and irreversibly to any molecule that has been biotinylated. This allows the creation of immunoadherent complexes without having to synthesize novel fused polypeptides or add synthetic linkers. Such experimental flexibility will facilitate the study of factors that affect the potency, distribution and metabolism of different FP-containing complexes *in vivo* in order to optimize their function as an accessory immunotherapeutic agents.

## Materials and Methods

### Fusion protein construction and purification

DNA for the fusion protein scFv (provided by Dr. James Atkinson, Washington Univ., St. Louis, MO) was fused in frame with coding sequences for core streptavidin (provided by Dr. Stephan Dubel, Technical Univ. of Braunschweig, Germany, based on Pahler, et al. [18]), followed by a polyhistidine sequence and inserted into pET 21a(+). BL21(DE3)*pLysS* cells (Invitrogen, Carlsbad, CA) were transformed with the resultant plasmid and expression of the recombinant protein was induced with IPTG. FP was purified from bacterial lysate using an SP-sepharose and a His-Select Nickel Affinity gel (Sigma-Aldrich) and eluted with TUB/100 buffer (60 mM Tris-HCl, 8 M urea, 100 mM imidazole, pH 8.0). FP was dialyzed in a series of buffers containing 50 mM Tris-HCl and 0.4 M arginine, slowly decreasing the urea concentration. The final preparation is in a buffer of PBS-Arg, pH 7.4 (5 mM NaH_2_PO_4_, 70 mM NaCl, 0.4 M arginine).

### Biotinylation and fluorescent labeling of mAbs

6A, 4LCA, and 13A antibodies were biotinylated using a FluoReporter Mini-Biotin-XX Protein Labeling Kit (Molecular Probes, Eugene, OR). HC50A, produced as in [21], was conjugated to Alexa488 using a DyLight 488 Antibody Labeling Kit and inactivated BoNT/A holotoxin (mBoNT/A-488), was labeled with a DyLight 488 Microscale Antibody Labeling Kit (Thermo Fisher Scientific, Rockford, IL).

### *in vitro* analysis of FP complexes binding to RBCs via flow cytometry

Heparinized RBCs collected from female Swiss Webster mice (Taconic Farms, Hudson, NY) were diluted 1∶2 in PBS:heparin (100 U/ml) and aliquoted at 10^6^ per well and washed with 200 µl PBSA (PBS/1% BSA). RBCs were incubated with or without 10-fold excess (4.4 mg) rat anti-mouse TER-119 or rat IgG2b isotype control (eBiosciences, San Diego, CA) and incubated at room temperature (RT) for 30 min. Cells were spun down at 2000 rpm in an Allegra 6R centrifuge with a GH3.8 rotor (Beckman-Coulter, Brea, CA) 5 min and incubated with 400 ng FP in 100 ml PBSA for 45 min. The mAb 6A, biotinylated or un-biotinylated, was incubated with A1-Alexa488 for 1 hr at RT, and then added to RBCs and incubated for 30 min. Cells were washed twice in PBSA and F(ab')2 donkey anti-human IgG APC (Jackson ImmunoResearch, West Grove, PA) added at 1∶10,000 and incubated at RT for 30 min. Cells were washed twice in PBSA and resuspended in a final volume of 1 ml PBSA and analyzed on a BD FACSCantoII (Becton Dickson, Franklin Lakes, NJ) using FlowJo 8.8.6. software (Tree Star, Ashland, OR). Competition experiments were performed as above by incubating 400 ng FP with biotin-fluorescein (Thermo Fisher Scientific) in 10-fold molar excess for 50 min, adding to RBCs pre-incubated with TER-119 as described previously for 30 min, washing twice and analyzing by FACS.

### Plasmid construction and bacterial expression of detoxified botulinum toxin type A (BoNT/A) protein

A codon-optimized BoNT/A igene was constructed (GenScript USA, Piscataway, NJ). The gene contained 4 point mutations, R363A and Y365F, which abolish the catalytic activity of BoNT/A [30], and W1266L and Y1267S, which prevent binding of BoNT/A at the neuromuscular junction [31]. The gene also encoded an N-terminal polyhistidine tag followed by an enterokinase site. The gene was inserted into the pTRCHisA vector, yielding the expression plasmid pTRC-detoxBoNT/A, and expressed in E. coli BL21-codon plus(DE3)-RIL (Agilent Technologies, Santa Clara, CA). Cells were grown in Terrific Broth (1.2% peptone, 2.4% yeast extract, 0.94% K2HPO4 and 0.22% KH2PO4) (Difco; Sparks, MD) at 37°C to ∼0.8 OD600, at which time IPTG was added, the culture was cooled to 20°C in an ice bath, and the cells were incubated, shaking, for 12 hours.

### Purification of protein

Bacterial cells from 1 liter of culture were suspended in 200 ml of bacterial protein extract reagent, B-PER (Pierce; Rockford, IL) at 4°C. Lysozyme (Sigma; St. Louis, MO) at a final concentration of 0.1 mg/ml, DNASE (Sigma) at a final concentration of 0.01 mg/ml, and protease inhibitor cocktail tablet (Roche; Manheim, Germany) were added to the cell suspension and incubated on a rotating shaker for 2 hr. Four hundred ml of 50 mM sodium phosphate containing 300 mM NaCl, pH 8.0, was added to the lysed cell suspension and allowed to stand for 30 min. The suspension was centrifuged at 27,000 x g for 40 min to remove precipitate.

The clear supernatant was loaded onto a 5 ml column of Ni-NTA superflow (Qiagen) which was equilibrated with 50 mM sodium phosphate containing 300 mM NaCl, pH 8.0. The column was washed with 50 volumes of washing buffer (50 mM sodium phosphate containing 300 mM NaCl, and 20 mM imidazole, pH 8.0). Bound protein was eluted from the column with a gradient of increasing imidazole (100 ml of 50 mM sodium phosphate containing 300 mM NaCl and 20 mM imidazole, and 100 ml of 50 mM sodium phosphate containing 300 mM NaCl and 250 mM imidazole, pH 8.0). The active fractions (at ∼80 mM imidazole) were pooled and dialyzed against 50 mM sodium phosphate, pH 6.8. The dialysate was centrifuged at 27,000 x g for 30 min to remove precipitate.

The clear supernatant was loaded onto a 4 ml cation exchange column of CM Sepharose fast flow (Amersham Bioscience; Piscataway, NJ) equilibrated with 50 mM sodium phosphate, pH 6.8. The column was washed with 50 volumes of 50 mM sodium phosphate, pH 6.8. Bound protein was eluted from the column with a stepwise increasing of NaCl concentration (10, 20, 40, 60, 100, 150, 200, 300 and 500 mM of NaCl with 50 mM sodium phosphate pH 6.8). The active fractions (at ∼200 mM NaCl) that correspond to about 150 kDa protein on a 10% SDS polyacrylamide gel electrophoresis were pooled and dialyzed against PBS. The purity of detoxified BoNT/A was confirmed on the SDS-PAGE and found to be more than 98% homogeneous. The identity of the BoNT/A was confirmed by Western blot analysis using rabbit polyclonal antibodies raised separately against the catalytic domain (LC) and the heavy chain domain (HC50) of pure BoNT/A toxin.

### Digestion with enterokinase and generation of dichain detoxified BoNT/A

C. botulinum produces BoNT/A as a di-chain active protein molecule (nicked). Recombinant detoxified BoNT/A purified from E. coli was treated with a protease enterokinase (EK) to make it a di-chain ‘nicked’ molecule. One milligram of purified detoxified BoNT/A was incubated with five units of enterokinase in a 1.5 ml of EK-Max buffer as described in EK-MaxTM kit (Invitrogen) for O/N at 25°C. EK was then removed by EK-away TM resign (Invitrogen). The digested protein sample was then diluted with 5 bed volumes of 50 mM sodium phosphate, 300 mM NaCl, and 10 mM imidazole (pH 8.0). The solution was centrifuged at 27,000 x g for 40 min to remove any precipitate.

The clear supernatant was passed through a 2 ml column of Ni-NTA superflow (Qiagen) equilibrated with 50 mM sodium phosphate, 300 mM NaCl, pH 8.0. The N-terminal polyhistidine tag that was cleaved of from the detoxified BoNT/A protein molecule was trapped by Ni-NTA resin and the remaining digested protein molecule passed through the column. The pass-through sample was dialyzed against PBS and concentrated using 30K ultracentricon (Millipore). The di-chain nicked detoxified BoNT/A was compared with the native BoNT/A by reducing SDS-PAGE and found to have two bands (a 50 kDA light chain and a 100 kDa heavy chain).

### Blood plasma BoNT ELISA determination

For measurement of clearance of BoNT from the circulation by FP, the detoxified BoNT/A was used. 6 µg of detoxified BoNT/A was incubated with 3 µg biotinylated 6A, 3 µg biotinylated 4LCA, and 24 µg FP for 1 hr at RT prior to tail vein injection in 25 g female Swiss Webster mice. Control mice were given toxin only or no injection. Mice were anesthetized under isofluorane 90 mins after toxin administration and whole blood was collected by cardiac puncture with a heparinized syringe. Blood was separated into microfuge tubes and spun for 5 minutes at 3,000 RPM in a microcentrifuge for 5 minutes. The plasma was aliquoted and stored at −20°C until use. To assay BoNT/A in plasma, black 96-well flat bottom (Nunc Maxisorp) plates were coated at 4°C overnight with 100 µl/well of 3B3, a human mAb that binds BoNT/A, at 2 µg/ml in PBS. Plates were washed with PBS/0.05% Tween-20 (Sigma-Aldrich, St. Louis) and then blocked for 1 h at 37°C with PBS/0.05% Tween-20/5% non fat dry milk. Undiluted samples were added at 100 µl/well, incubated for 2 hours at 37°C, washed, and followed by addition of rabbit anti-HC50A serum at 1∶100 dilution. After one hour, goat anti-rabbit-HRP was used at 100 µl/well (1∶10,000 dilution) and incubated for an additional hour. OPD was used as the colorimetric substrate; optical density at 490 nm was measured.

### Botulinum neurotoxins

BoNT/A1 and BoNT/B were obtained from Metabiologics (Madison, WI). LD50 eqivalents were 2.5 pg/LD50 for BoNT/A1 and 5 pg/LD50 for BoNT/B.

### Ethics statement

All animal work was conducted according to all relevant guidelines in a protocol approved by the Institutional Animal Care and Use Committee of the Lankenau Institute for Medical Research, covered by protocol number A08-2692, Approval Date: August 26, 2008, last amendment approval date, July 15, 2009, Animal Welfare Assurance number A3550-01.
